# Inflammation and Negative Symptoms of Schizophrenia: Implications for Reward Processing and Motivational Deficits

**DOI:** 10.3389/fpsyt.2020.00046

**Published:** 2020-02-20

**Authors:** David R. Goldsmith, Mark Hyman Rapaport

**Affiliations:** Department of Psychiatry and Behavioral Sciences, Emory University School of Medicine, Atlanta, GA, United States

**Keywords:** inflammation, cytokines, negative symptoms, reward, motivation, schizophrenia

## Abstract

Negative symptoms of schizophrenia are debilitating and chronic in nature, are difficult to treat, and contribute to poor functional outcomes. Motivational deficits are a core negative symptom and may involve alterations in reward processing, which involve subcortical regions such as the basal ganglia. More specifically, dopamine-rich regions like the ventral striatum, have been implicated in these reward-processing deficits. Inflammation is one mechanism that may underlie negative symptoms, and specifically motivational deficits, via the effects of inflammatory cytokines on the basal ganglia. Previous work has demonstrated that inflammatory stimuli decrease neural activity in the ventral striatum and decrease connectivity in reward-relevant neural circuitry. The immune system has been shown to be involved in the pathophysiology of schizophrenia, and inflammatory cytokines have been shown to be altered in patients with the disorder. This paper reviews the literature on associations between inflammatory markers and negative symptoms of schizophrenia as well as the role of anti-inflammatory drugs to target negative symptoms. We also review the literature on the role of inflammation and reward processing deficits in both healthy controls and individuals with depression. We use the literature on inflammation and depression as a basis for a model that explores potential mechanisms responsible for inflammation modulating certain aspects of negative symptoms in patients with schizophrenia. This approach may offer novel targets to treat these symptoms of the disorder that are significant barriers to functional recovery and do not respond well to available antipsychotic medications.

## Negative Symptoms: A Focus on Motivational Deficits and Reward Processing

Schizophrenia is a severe mental illness that affects 1% of the population and accounts for over $60 billion in US healthcare costs (1, 2). Up to 30% of individuals with schizophrenia are considered “treatment-resistant,” adding to even greater morbidity and socioeconomic burden (3, 4). The disorder is a major public health concern, and many people with schizophrenia suffer chronic debilitating symptoms (5, 6), have high rates of unemployment and homelessness in the US (7, 8), and have a significantly reduced life expectancy (9).

In contrast to delusions and hallucinations that constitute the positive symptoms of schizophrenia, negative symptoms characterize absent or diminished behavior and include motivational deficits, social withdrawal, poverty of speech, decreased emotional reactivity and psychomotor expression, and deficits in volition (10, 11). Negative symptoms are common and are thought to be present in over half of patients, with reports in the literature ranging from 60 to 90% (12, 13). These negative symptoms may be primary in nature (i.e., thought to be driven by underlying pathophysiology of the disorder). Alternatively, they may be secondary to other causes, including positive symptoms (i.e., amotivation to leave the house secondary to paranoid delusions), depression, anxiety, or side effects of medications (i.e., lack of facial affect secondary to extrapyramidal symptoms) (14). The literature has also described a subtype of schizophrenia referred to as “deficit schizophrenia,” which is marked by primary and enduring negative symptoms, and is thought to occur in ~20% of individuals with schizophrenia (15). Negative symptoms are common in individuals with treatment resistant schizophrenia, and may explain the most variance in disease severity and mediate the relationship between disease severity and cognitive deficits in this patient population (16, 17). Importantly, negative symptoms are consistently shown to be predictive of functional impairment and poor outcome in patients with schizophrenia, more so than positive symptoms of the disorder (18–22). Current antipsychotics do not adequately treat negative symptoms, which remain a distinct challenge in the treatment and management of patients with schizophrenia (23, 24).

Impaired reward processing and motivational deficits are the negative symptoms most predictive of poor quality of life and functional impairment (11, 25–28): patients often fail to expend effort to seek out rewarding activities such as work and social interaction. These motivational deficits can be further delineated into four measurable domains: decreased reward anticipation, impaired reinforcement learning, reward prediction errors, and reduced effort-cost computation (29). It is also important to note that given the current focus of the National Institute of Mental Health to discover mechanisms underlying dimensions of behavior using a transdiagnostic approach (Research Domain Criteria, RDoC), decreases in motivation and reward processing seen in negative symptoms of schizophrenia as well as depression fall under the RDoC domain of Positive Valence Systems (30–32).

Subcortical brain regions including the basal ganglia, and specifically the ventral striatum, are part of a distributed brain network that subserves all of these domains, which also include the ventral tegmental area, prefrontal cortex, and anterior cingulate cortex among others (33, 34). Neuroimaging studies demonstrate reduced ventral striatal responses to reward anticipation (35–38), reinforcement learning (39, 40), and positive prediction errors for patients with schizophrenia (41–44). Patients with schizophrenia also have decreased objective reward processing as measured by assessments of effort expenditure for reward and reinforcement learning (45–56), which are known to involve ventral striatal circuits (35–37, 48, 57–59). Decreased activity in the ventral striatum on a monetary reward based neuroimaging task has been directly related to both objective and clinical assessments of decreased motivation in patients with schizophrenia (48). Decreased ventral striatal activity and task performance have been consistently related to severity of negative symptoms (39, 46, 48, 51, 53, 54, 56, 60). Moreover, patients with treatment resistant schizophrenia with significant negative symptom severity, may have different neural reward processing responses relative to those who are treatment-responsive (61). These converging findings suggest that reward processing deficits are a core component of negative symptoms in schizophrenia.

Heterogeneity in clinical presentation and etiology of schizophrenia presents a challenge to discovering pathways and mechanisms that underlie the complex symptoms of the disorder (62–64). Despite the challenges of disentangling this heterogeneity, understanding mechanisms that may underlie symptoms of the disorder are important to consider and investigate. Inflammation may represent one pathophysiologic mechanism that underlies motivational and reward processing deficits in patients with schizophrenia. Although multiple aspects of the immune system have been implicated in the pathophysiology of schizophrenia, we will focus on the role of inflammation. Our previous meta-analysis (65) demonstrated alterations in peripheral inflammatory markers in patients with not only acute exacerbations of symptoms of schizophrenia, but also in chronically ill patients with schizophrenia where negative symptoms tend to be most prominent. This paper presents the evidence for the role of inflammation in schizophrenia, reviews data showing associations between inflammatory markers and negative symptoms, and presents data from treatment trials with anti-inflammatory medications. This review will also discuss data showing relationships between inflammatory cytokines and motivational deficits in other psychiatric disorders that may provide a framework for studying these relationships in patients with schizophrenia. Finally, we will propose future directions to drive forward research investigating novel mechanisms that may be responsible for negative symptoms and motivational deficits in some patients with schizophrenia.

## Multiple Lines of Evidence Implicate Inflammation and the Immune System in the Pathogenesis of Schizophrenia

Epidemiological studies demonstrated that exposure to infections *in utero* and in childhood increases the risk for schizophrenia (66–68). Autoimmune conditions are more prevalent in both individuals with schizophrenia and their first-degree relatives (69, 70). Moreover, genome-wide association studies have repeatedly shown an association between schizophrenia and immune genes, including the major histocompatibility complex region on chromosome 6 (71–73). These findings are consistent with data that the complement pathway (innate immune system) may play a fundamental role in the development and progression of the syndrome *via* effects on synaptic pruning (74). In a meta-analysis, we reported that patients with schizophrenia reproducibly exhibit alterations in peripheral inflammatory marker concentrations (65), and several of the included studies reported associations between cytokines and negative symptoms (75–80).

## Insights Into the Effects of Inflammation on Negative Symptom of Schizophrenia

A number of groups have reported associations between inflammatory markers and negative symptoms of schizophrenia, although many of these studies have only investigated a single inflammatory marker. Another of the challenges with these studies has been the focus on a variety of different stages of illness. Moreover, the majority of these analyses are correlative in nature. Thus it is challenging to formulate a coherent understanding of the role of inflammation in the pathogenesis of negative symptoms. **Table 1** summarizes these findings that report an association between inflammation and negative symptoms.

**Table 1:** Description of studies showing associations between inflammatory markers and negative symptoms of schizophrenia.

	Markers studied	Serum/plasma	Assay	Population studied	N	Factors controlled for/included in statistical models	Comments
Fan et al. (81)	CRP	Serum	Particle enhanced immunonephelometry	Inpatients with schizophrenia/schizoaffective disorder; no control group	26	None	High CRP group (>0.5 mg/dl; n=5) had higher PANSS scores on all subscales, including negative symptoms
Boozalis et al. (82)	CRP	Plasma	ELISA	Inpatients with schizophrenia; no control group	39	Age, sex, race, BMI	Positive correlation between CRP and PANSS negative symptoms both unadjusted and after adjusting for age, sex, race, and BMI
Liemburg et al. (83)	CRP	Plasma and Serum collected from different sites	Varied by sites; specific assays not disclosed	Outpatients from four different sites in the northern Netherlands; no control group	2123	Age, sex, smoking, use of anti-histaminergic antipsychotics, statins, fibrates, corticosteroids, antibiotics, chlorpromazine equivalents, BMI, metabolic syndrome, metabolic effects of antipsychotics (high, medium, low)	Association between CRP and PANSS negative symptom subscale in linear regression models
Garcia-Rizo e al. (75)	CRP and IL-6	Not described	IL-6: ELISA CRP: not described	Antipsychotic naïve patients with first episode nonaffective psychosis; no control group	20 patients with deficit psychosis and 42 patients with non-deficit psychosis	Groups matched for age, sex, BMI, smoking	Higher concentrations of CRP and IL-6 in the deficit group compared to the non-deficit group
Goldsmith et al. (84)	IFN-γ, IL-1β, IL-6, sIL-2R, TNF	Plasma	Multiplex immunoassay	Outpatients with schizophrenia and healthy controls	17 with deficit schizophrenia, 39 with non-deficit schizophrenia, 28 controls	Smoking, BMI, education	Higher concentrations of IL-6 and TNF in deficit patients compared to non-deficit and controls. TNF associated with PANSS negative symptoms in linear regression models
Stojanovic et al. (77)	IL-6, CRP, fibrinogen	Serum	CRP by immunoturbidimetry assay; IL-6 by ELISA	Outpatients with psychotic disorder (PD), ARMS subjects, healthy controls	77 with psychotic disorder, 17 ARMS subjects, 25 controls	Sex, BMI, substance use, antipsychotic treatment, IL-6 rs1800795 genotype	Higher concentrations of IL-6 in ARMS compared to control group and in PD compared to control that becomes trend-level after Bonferroni correction. CRP differences between groups do not meet significance after Bonferroni correction. IL-6 associated with negative symptoms in linear regression models for both PD and ARMS subjects
Goldsmith et al. (85)	IFN-γ, IL-1β, IL-1RA, IL-4, IL-6, IL-8, IL-10, TNF	Plasma	Multiplex Immunoassay	CHR subjects; no control group	37	Age, sex, race, weight, baseline negative symptoms, baseline CDSS scores	Higher concentrations of TNF and lower concentrations of IL-6 predicted worse negative symptom trajectories at one year follow up
Xiu et al. (78)	IL-10	Serum	ELISA	First episode drug naïve inpatients with schizophrenia; heathy controls	128 patients with schizophrenia; 62 controls	Sex, age, education, smoking, BMI	Decreased IL-10 concentrations in the patients compared to controls. IL-10 was inversely correlated with negative symptoms severity on the PANSS.
Zhu et al. (86)	TNF and IL-1β	Serum	ELISA	First episode drug naïve patients with schizophrenia (both in and outpatients), chronic patients with schizophrenia (both in and outpatients), and healthy controls	69 first episode patients, 87 patients with chronic schizophrenia, 61 healthy controls	Age, sex, course of illness	TNF and IL-1β concentrations were lower in first episode patients compared to healthy controls and higher in chronic patients compared to controls. Concentrations of both were correlated with the PANSS negative subscale in chronic, but not first episode patients.
Asevedo et al. (76)	IL-2	Plasma	Cytometric bead array	Outpatients with chronic schizophrenia and healthy controls	29 patients with schizophrenia; 26 controls	Differences between clozapine and other atypical antipsychotics was assessed	IL-2 concentrations were lower in patients compared to controls. IL-2 concentrations were negatively correlated with PANSS negative subscale score
Bresee et al. (87)	sIL-2R	Serum	ELISA	Outpatients with schizophrenia and healthy controls	59 patients with schizophrenia; 57 controls	Sex, age, smoking, BMI, type of pharmacotherapy	sIL-2R concentrations were elevated in patients compared to controls. sIL-2R concentrations were correlated with PANSS negative subscale score
El Kissi et al. (79)	IFN-γ,IL-4, TGF-β, IL-17, BAFF	Serum	ELISA	Antipsychotic free acute inpatients with schizophrenia and healthy controls	60 patients with schizophrenia; 28 controls	None	Positive correlation between IFN-γ and SANS total score; Negative correlation between IL-17 and SANS total score
Noto et al. (80)	CCL11, CCL24, MCP-1, MIP-1α, IL-8, IP-10, sTNF-R1, sTNF-R2, TNF, IL-2, IL-4, IL-6, IL-10, IFNγ, IL-17	Serum	ELISA	Outpatients with schizophrenia and healthy controls	54 patients with schizophrenia, 118 healthy controls	Sex, age, BMI, smoking, drug/alcohol use, ethnicity, monthly income (but not controlled for in all analyses)	Negative correlation between IL-2 and PANSS negative subscale score; Positive correlation between CCL11 and PANSS negative score

CRP, C-Reactive Protein; PANSS, Positive and Negative Syndrome Scale; BMI, body mass index; ELISA, enzyme-linked immunosorbent assay; CDSS, Calgary Depression Scale for Schizophrenia; IL-6, interleukin 6; IFN-γ, interferon gamma; IL-1β, interleukin 1 beta; sIL-2R, soluble interleukin 2 receptor; TNF, tumor necrosis factor; ARMS, at risk mental state; IL-1RA, interleukin 1 receptor antagonist; IL-4, interleukin 4; IL-8, interleukin 8; IL-10, interleukin 10; CHR, clinical high risk; IL-2, interleukin 2; TGF-β, transforming growth factor beta; IL-17, interleukin 17; BAFF, B cell activating factor of the tumor necrosis factor family; SANS, Scale for the Assessment of Negative Symptoms; CCL11, eotaxin-1; CCL24, eotaxin-2; MCP-1, monocyte chemoattractant protein-1; MIP-1α, macrophage inflammatory protein 1α; IP-10, interferon-γ-inducible protein 10; sTNF-R1, soluble tumor necrosis factor receptor 1; sTNF-R2, soluble tumor necrosis factor receptor 2.

A few studies have investigated the role of C-reactive protein (CRP), an acute phase protein synthesized by the liver that can be induced by cytokines (88, 89), and has been shown to be elevated in patients with schizophrenia in multiple meta-analyses (90, 91). Early work (81) demonstrated that inpatients with schizophrenia (n = 5) with CRP > 0.5 mg/dl had higher scores on the Positive and Negative Syndrome Scale (PANSS) negative symptom score than those inpatients with schizophrenia with CRP< 0.5 mg/dl (n = 21). The high CRP group had higher scores on all subscales (and total score) of the PANSS, which could reflect the effect of the stress of acute psychosis stimulating an inflammatory response rather than a specific interaction between inflammation and negative symptoms. Boozalis *et al*. found a modest relationship between CRP and negative symptoms as assessed by the PANSS in a small sample (n = 39) of patients with schizophrenia that remained significant after adjusting for age, sex, race, and body mass index (BMI) (82). Similarly, Liemburg *et al*. found that CRP was correlated to PANSS negative score (as well as positive score) in a large sample (n = 2,132) of outpatients with chronic schizophrenia in the Netherlands (83).

Inflammatory markers such as CRP have been elevated in patients with deficit schizophrenia, which is a term used to describe patients who suffer mostly primary and enduring negative symptoms despite attempts to optimize medication interventions (14, 92). In a study of deficit compared to non-deficit patients, both CRP and interleukin (IL)-6 concentrations were higher in the deficit group (75). Goldsmith et al. extended this work, finding higher concentrations of the pro-inflammatory cytokines tumor necrosis factor (TNF) and interleukin (IL)-6 in deficit patients compared to both non-deficit patients and healthy controls (84). Moreover, TNF (but not IL-6) was associated with PANSS negative scores (and not other subscale scores) in linear regression models.

Other studies have examined the relationship between a number of different inflammatory cytokines and negative symptoms across the spectrum of psychosis, from individuals at clinical high risk to first episode to chronic schizophrenia. Stojanovic and colleagues found that increased concentrations of IL-6 were associated with higher negative symptom scores on the PANSS in both individuals determined to be at an at-risk mental state as well as young individuals diagnosed with a psychotic disorder (77). In a study of clinically high risk individuals from the North American Prodromal Longitudinal Study (NAPLS), higher concentrations of TNF predicted worse negative symptom trajectories at 1 year follow up whereas lower IL-6 concentrations predicted worsening negative symptoms trajectories (85). Although it is unclear why the findings for TNF and IL-6 diverged, one possible explanation may have to do with the pleiotropic nature of IL-6 in the immune response (93, 94). For example, IL-6 may exert its pro-inflammatory effect via trans-signaling with its soluble receptor, whereas IL-6 may also have anti-inflammatory properties via classical signaling pathways involving membrane-bound IL-6 receptors and the signal transducing beta-subunit glycoprotein 130 (gp130) (95, 96).

One study has examined the relationship between inflammatory biomarkers and negative symptoms in first episode psychosis. Xiu et al. found that drug naïve first episode patients had decreased concentrations of IL-10, an anti-inflammatory cytokine, relative to matched controls, and IL-10 was inversely correlated with negative symptoms severity on the PANSS (78). Interestingly, recent work from Zhu *et al*. showed elevated TNF-a and IL-1beta in patients with chronic schizophrenia compared to healthy controls, whereas drug naïve first episode patients had lower concentrations compared to both the chronic patients and controls (86). Both TNF and IL-1beta were correlated with PANSS negative subscale scores in chronic patients, but not in the first-episode cohort.

Other studies of chronic patients with schizophrenia have also shown relationships between cytokines and cytokine receptors and negative symptoms. Asevedo *et al*. found that individuals with chronic schizophrenia had lower concentrations of IL-2 compared to controls, and IL-2 was negatively correlated with PANSS negative symptoms scores (76), consistent with findings from Bresee and Rapaport who demonstrated that soluble IL-2 receptor (sIL-2r) was correlated with PANSS negative, general, and total scores (87).

All of the above studies (save for the two from Goldsmith et al.) measured single inflammatory markers, which may not provide a full understanding of which inflammatory markers may play a role in negative symptoms. Moreover, the measurement of a uniform panel of inflammatory markers would allow for comparison of markers across studies and inform the field's understanding of the overall patterns of immune activation relative to these symptoms (65). Two studies in patients with schizophrenia investigated more than one or two individual markers. El Kissi *et al*. measured five inflammatory/immune markers in acute drug-free patients with schizophrenia and found a positive correlation between interferon-gamma and negative symptoms on the Scale for the Assessment of Negative Symptoms (SANS) and a negative correlation between IL-17 and SANS scores (79). Noto and colleagues measured 15 inflammatory/immune biomarkers and found a significant negative relationship between PANSS negative score and IL-2 and a positive association with the chemokine CCL11 (80).

## Negative Symptoms and Anti-Inflammatory Treatment Trials

There is a growing literature on the role of anti-inflammatory treatments in individuals with schizophrenia (97–99). Given the heterogeneity of inflammatory markers in the studies demonstrating relationships with negative symptom severity, blocking inflammation in treatment trials represents a complementary approach to understanding these relationships. The most well-studied anti-inflammatory medications have been the non-steroidal anti-inflammatory medications, including COX-2 inhibitors and aspirin. For example, the COX-2 inhibitor celecoxib showed significant benefit for negative symptoms (in addition to positive and general symptom scores on the PANSS) as an add-on treatment to antipsychotics in a number of studies (100, 101). Results from meta-analyses have been mixed depending on which studies are included. For example, Muller *et al*. found benefit for the cyclo-oxygenase-2 (COX-2) inhibitor celecoxib as an adjuvant treatment with the antipsychotic amisulpride on negative symptoms in early stage schizophrenia (101), whereas Rapaport *et al*. found no such benefit for celecoxib in chronically ill patients treated with either olanzapine or risperidone (102). In meta-analyses, Sommer *et al*. found a significant benefit for nonsteroidal anti-inflammatory drugs (NSAIDS) for negative symptoms across five studies (103), whereas Nitta *et al*. did not find a significant benefit for negative symptoms (104). A more recent meta-analysis has demonstrated a significant benefit for NSAIDS in first-episode patients, but not individuals with chronic schizophrenia (105).

Minocycline, a tetracycline antibiotic that has been shown to purportedly be neuroprotective *via* it's anti-inflammatory and anti-apoptotic properties (106), has also been investigated in patients with schizophrenia. A recent meta-analysis demonstrated benefit for minocycline across eight randomized controlled trials (107), although the largest study to date showed no benefit for minocycline for negative symptoms, which was the primary outcome for the study (108). The heterogeneity in results across studies (both individual and meta-analyses) for NSAIDs and minocycline may suggest that there are some individuals who would benefit from anti-inflammatory medications. Individuals with elevated inflammation may represent such a group, as has shown to be the case in patients with depression (109–111), though this was not the case in the recent minocycline trial (108).

## The Role of Inflammation in Reward Processing Deficits in Psychiatric Illness: Insights Into Negative Symptoms

Deficits in reward processing and decreased motivation have been consistently shown to be present in various psychiatric disorders, including major depressive disorder, bipolar disorder, as well as schizophrenia (112), which have all been shown to have altered peripheral inflammatory marker concentrations (65). The effect of peripheral inflammatory cytokines on the ventral striatum and other regions of the basal ganglia has been linked to deficits in reward processing and decreased motivation (113). Peripheral inflammation alters neural activity in ventral striatal regions following administration of several inflammatory stimuli including interferon (IFN)-alpha, typhoid vaccination, and endotoxin (113–116). Increased inflammation mediates deficits in effort expenditure in studies of laboratory animals and non-human primates (117–120). Functional magnetic resonance imaging (fMRI) of subjects with major depressive disorder (MDD) and increased inflammation (as measured by peripheral C-reactive protein; CRP) has been associated with decreased functional connectivity between ventral striatum and ventromedial prefrontal cortex (121). Decreased connectivity between these regions was correlated with decreased motivation and increased peripheral levels of interleukin (IL)-6 and IL-1 receptor antagonist (IL-1RA). The TNF antagonist infliximab has been shown to markedly reduce inflammatory marker concentrations and symptoms of depression, including motivational deficits, in people with major depression with increased inflammation (> 5 mg/L) (109).

**Figure 1:**
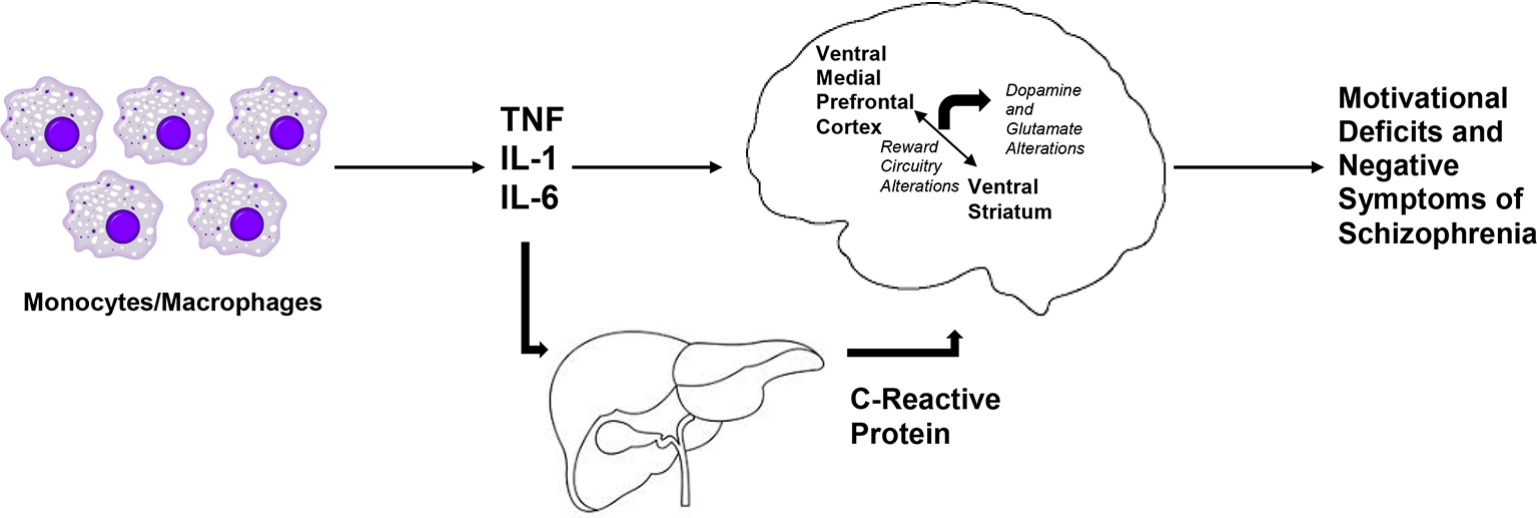
Hypothesized relationship between inflammatory cytokines and negative symptoms of schizophrenia. We hypothesize that markers of inflammation of monocytic origin, such as tumor necrosis factor (TNF), interleukin 1 (IL-1), and interleukin 6 (IL-6) are increased in patients with schizophrenia. Along with the acute phase reactant, C-Reactive Protein (CRP), these inflammatory markers access the brain to lead to decreased activation of the ventral striatum and decreased connectivity in reward-relevant regions of the brain, such as between the ventral striatum and the ventral medial prefrontal cortex. Together with subsequent dysfunction in dopaminergic and glutamatergic signaling, increased inflammation may lead to motivational deficits and negative symptoms in patient with schizophrenia.

Patients with schizophrenia who exhibit motivational deficits (52, 60), show decreased activation of the ventral striatum to reward anticipation in fMRI tasks, and decreased activation in the ventral striatum has been shown to be inversely correlated with negative symptom severity (36). We believe that these findings as well as the corelative data discussed above suggests that there is an opportunity to employ a transdiagnostic approach studying the effects of inflammation on reward processing and negative symptoms. Recent evidence from Park and colleagues using task-based fMRI begins to identify both similarities and differences in the neural circuit responses to an effort-based reinforcement task for patients with major depression and schizophrenia (122). One transdiagnostic finding that has been similar for subjects with schizophrenia and major depression has been the positive relationship between dopamine transporter availability and the fMRI BOLD response in the nucleus accumbens to reward anticipation has been described in both disorders (123). Individuals at clinical high-risk for schizophrenia from the North American Prodrome Longitudinal Study (NAPLS) cohort have depressive symptoms that have been shown to be associated with negative symptoms (124). This association has also been reported in longitudinal data of patients with schizophrenia extending from the first episode to 10+ years of chronic psychosis (125). Furthermore, a recent meta-analysis reported a relationship between negative symptoms of schizophrenia and defeatist personal beliefs, a cognitive construct thought to underlie motivational deficits in individuals with depression (126). In summary, neural mechanisms underlying motivational deficits may be similar in depression and in negative symptoms of schizophrenia, and we hypothesize that these findings may reflect the impact of inflammation on reward circuitry across diagnoses (see **Figure 1**).

## Inflammation, Dopaminergic/Glutamatergic Signaling, and Reward Circuitry Alterations in Schizophrenia

It is important to note that the majority of studies (including all studies listed in **Table 1**) measure inflammatory markers in peripheral blood, which raises the question: do similar inflammatory changes also occur centrally in the brain? This is especially important given that inflammatory markers such as CRP and cytokines are large molecules (~15–25 kD) and are not freely able to cross the blood brain barrier (BBB). These markers are able to access the brain to activate local inflammatory markers via a number of different mechanisms including 1) movement through leaky parts of the BBB, 2) active uptake via transporter systems, 3) activation of endothelial and immune cells in cerebral vasculature and subsequent release of inflammatory markers in the brain, and 4) via peripheral afferents, such as the vagus nerve, that may relay cytokine signals to the brain (127–129). One piece of evidence that suggests similar changes in inflammatory marker concentrations are occurring centrally comes from studies measuring cerebrospinal fluid (CSF) which demonstrate similar patterns in inflammatory marker concentrations in patients with schizophrenia relative to healthy controls (130, 131). Recent evidence in patients with major depressive disorder demonstrates that peripheral CRP and inflammatory cytokines are strongly correlated with CSF markers, which suggests that peripheral markers may reflect similar findings in the central nervous system (132).

Inflammation leads to decreases in dopamine release and increased glutamate activity in some patients with depression and this has been correlated with reward processing and motivational deficits (112, 133). Inflammation impacts dopaminergic and glutamatergic signaling *via* a variety of potential mechanisms (134). Inflammation decreases dopamine availability and dopamine release (135), and may decrease concentrations of tetrahydrobiopterin, a necessary co-factor in dopamine synthesis (136). Similarly, proinflammatory cytokines decease glutamate transporter expression on the cell surface of astrocytes and induce glutamate release. Proinflammatory cytokines also modulate the kynurenine pathway, leading to increased quinolinic acid release from microglia, which binds to N-methyl-D-aspartate (NMDA) receptors and can stimulate glutamate release and block reuptake from astrocytes (137).

Dopaminergic and glutamatergic dysfunction has been postulated to be central to the underlying pathophysiology of schizophrenia, with putative hyperdopaminergic states in subcortical regions as well as NMDA-receptor hypofunction (138–140). Dopaminergic and glutamatergic signaling have also been consistently implicated in reward processing and effort cost computation in healthy subjects and individuals with schizophrenia (29, 46, 53, 141–145). Evidence suggests downstream effects of dopamine and glutamate on connectivity in brain circuitry in patients with schizophrenia (146, 147) including disrupted resting state networks (148, 149).

As expected in a heterogeneous syndrome such as schizophrenia and given the complex circuit-dependent actions of neurotransmitters, some studies (150, 151), have demonstrated that individuals with treatment resistant schizophrenia, who may have persistent negative symptoms (16, 152), may have decreased dopamine synthesis capacity. This is further supported by recent evidence that phase of illness and antipsychotic medications may alter dopaminergic tone by increasing presynaptic dopamine capacity (153). As such, there may be heterogeneity in dopamine signaling pathways such that the underlying hyperdopaminergic state in subcortical regions, which was long thought to be the field's understanding of dopamine signaling in patients with schizophrenia (154), may only be present in a subgroup of patients. Other patients may have hypodopaminergic signaling in these regions, as reflected by decreased presynaptic dopamine synthesis capacity. These patients may also have different glutamatergic signaling profiles suggestive of a different underlying neurobiology. In fact, there is recent evidence demonstrating increased glutamatergic signaling in the anterior cingulate of treatment resistant patients with schizophrenia compared to antipsychotic responsive patients with schizophrenia (155). Thus, there seems to be a complex and possibly circuit specific interplay between dopamine, glutamate, and potentially other neurotransmitters. It is possible that known effects of inflammation on dopamine and glutamate signaling could be responsible for at least one of the different neurotransmitter signaling profiles that have been described in patients with schizophrenia. Future work will be necessary to test this hypothesis as it relates to inflammation and dopamine/glutamate signaling in patients with schizophrenia.

## Inflammation and Negative Symptoms: Future Directions

Despite effective treatments that target positive symptoms of schizophrenia, the treatment of negative symptoms remains a challenge; this is particularly important since negative symptoms are responsible for significant disability and poor function (24, 156, 157). Multiple pathophysiologic mechanisms outside of traditional dopamine-2 antagonism have been studied in an attempt to palliate negative symptoms, including alpha-7 nicotinic agonists/partial agonists, D-amino acid oxidase inhibitors, NMDA receptor glycine site antagonists, glycine transporter 1 inhibitors, mGluR2/3 positive allosteric modulators, muscarinic acetylcholine agonists, and amphetamine based compounds, with limited success (24). More recent strategies to target negative symptoms include modulating cyclic guanosine monophosphate (cGMP) (158), trace amine-associated receptor 1 (TAAR1) agonists (159), cannabidiol (160) but are in early phases of development (161).

Some of these novel strategies may involve pathways that impact the immune system. Given increasing recognition of the role that the immune system plays in schizophrenia (162), future work should seek to understand the mechanism by which the immune system, and specifically inflammation, may impact the brain to lead to negative symptoms of schizophrenia. Work in depression may serve as a model to test hypotheses regarding these mechanisms. For example, much of the work in depression has focused on inflammation's impact on anhedonia and motivational deficits. Although both are core negative symptoms in patients with schizophrenia, it is unclear whether other negative symptoms, such as deficits in affect may also be driven by inflammatory mediators. Future work may be aided by using some of the more recently developed negative symptoms scales, such as the Brief Negative Symptom Scale (163) or the Clinical Assessment Interview for Negative Symptoms (164), which have separate subscales that reflect deficits in affect and deficits in motivation. These scales may allow for the greater differentiation of negative symptoms from depression. Moreover, scales that directly address motivation, such as the Motivation and Pleasure Scale (165) may be a more specific outcome measure. Similarly, behavioral tasks that directly assess effort-based motivation (166) have been shown to be sensitive to the effects of inflammation (167) as well to deficits and negative symptoms in patients with schizophrenia (47, 53, 55, 168).

In order to better investigate relationships between inflammation and negative symptoms, the field must move away from simple correlations between measured inflammatory markers and negative symptoms. Due to the heterogeneity in clinical presentations of schizophrenia, focusing on those symptoms that are known to be targeted by the effects of inflammation would represent a hypothesis-driven approach. The impact of peripheral inflammation on the brain appears to be an evolutionarily conserved process (169) *via* effects on the basal ganglia, dopamine signaling, and subsequent motivational deficits. Focusing on these deficits and on the impact of inflammation on the basal ganglia using neuroimaging strategies could provide evidence suggesting that a similar process occurs in patients with persistence of negative symptoms of schizophrenia. For example, studies may choose to compare patients with high *versus* low inflammation [i.e., CRP cutoffs based on American Heart Association/Center for Disease Control and Prevention guidelines (170)] or high *versus* low negative symptom severity. This may partially explain seemingly negative findings from treatment trials using cytokine antagonists, such as a recent study that showed no benefit from tocilizumab, an anti-IL-6 receptor antagonist (171). Approaches such as stratifying patients based on a marker such as CRP, as has been done in depression (109, 111, 172), should be considered in treatment trials in patients with schizophrenia. Moreover, in order to disentangle heterogeneity, considering phase of illness will be important as well. Given known differences in inflammatory marker concentrations in individuals at clinical high risk, first episode psychosis, acute psychosis, and chronic schizophrenia, studying a heterogeneous group of patients who are at different phases of illness may obscure important biological findings (65, 173–175). Similarly, designing longitudinal studies to measure inflammatory cytokines over time and phase of illness are essential for investigating the trajectory of negative symptom and functioning.

Another important consideration is the need for agreement about which inflammatory markers to measure and what assay platform to use: this would facilitate greater reproducibility and comparison across studies. Studying patterns of immune activation with a uniform panel of markers also allows the field to create more reliable ratios of pro-inflammatory to anti-inflammatory markers (e.g., TNF : IL-10) which may provide more useful insights into the role these cytokines play in the pathophysiology of the symptoms compared to individual cytokines alone.

Increasingly, inflammatory markers such as IL-6, largely thought to be pro-inflammatory, has been shown to be pleiotropic in nature and may even have anti-inflammatory properties (176). Furthermore, individual differences in the expression and regulation of these inflammatory markers is complex and may lead to different behavioral effects (177, 178). The field must also consider and agree upon a common set of variables to control for in analyses that could confound the relationship between inflammation and brain regions/circuits and behavior. For example, sex, BMI, or other measures of insulin resistance, smoking, illicit drug use, antipsychotic exposure, stage of illness, and education or other proxy of socioeconomic status have all been shown to be associated with alterations in inflammatory molecules (65, 179). Variables related to metabolism/insulin resistance are important and have been demonstrated to be important even for drug-naïve first episode and clinical high-risk individuals who have been shown to have alterations in metabolic markers at the first episode (180). Collaborations with immunologists may help us better understanding the role these markers play and allow for more nuance in interpretation of the growing data in this field.

Outstanding questions also remain regarding how inflammation may impact dopaminergic and glutamatergic systems. In healthy controls exposed to inflammatory stimuli and in patients with depression, inflammation appears to decrease dopaminergic signaling (112, 115, 181) and increased glutamate in subcortical regions (133, 182). How these putative mechanisms alter known dopaminergic and glutamatergic abnormalities in patients with schizophrenia is an important question that must be addressed. Furthermore, understanding the interplay of the immune system with metabolism (180, 183, 184) or the kynurenine pathway (185, 186) may offer an approach to understand the complexities of how the immune system may impact the brain in patients with schizophrenia to lead to negative symptoms. Strategies such as challenges with drugs that target inflammation and/or these neurotransmitter systems, neuroimaging approaches such as positron emission tomography (PET) or magnetic resonance spectroscopy (MRS) or perhaps using novel approaches such as induced pluripotent stem cells (iPSC) from patients with schizophrenia may help elucidate these mechanisms.

Novel approaches to understand and treat negative symptoms of schizophrenia are of paramount importance. The immune system has been implicated in the pathophysiology of schizophrenia and previous studies of psychopathology and anti-inflammatory trials offer clues to the possibility that the immune system may underlie negative symptoms of the disorder. The role of the immune system in depression offers important and intriguing hypotheses as to the mechanism behind inflammations and negative symptoms *via* its impact on basal ganglia regions and neurotransmitter systems. Similar approaches should be undertaken in patients with schizophrenia to investigate whether these mechanisms are transdiagnostic. Given the burden of negative symptoms on patients with schizophrenia, further understanding the impact of the immune system on these symptoms is of great necessity.

## Author Contributions

DG conducted the literature review. Both DG and MR conceptualized, wrote, and edited the manuscript.

## Funding

DG is supported by a NIMH K23 MH114037, NIMH L30 MH114414.

## Conflict of Interest

The authors declare that the research was conducted in the absence of any commercial or financial relationships that could be construed as a potential conflict of interest.
